# Integrating Biological Covariates into Gene Expression-Based Predictors of Radiation Sensitivity

**DOI:** 10.1155/2017/6576840

**Published:** 2017-02-08

**Authors:** Vidya P. Kamath, Javier F. Torres-Roca, Steven A. Eschrich

**Affiliations:** ^1^Department of Biostatistics & Bioinformatics, H. Lee Moffitt Cancer Center & Research Institute, Tampa, FL, USA; ^2^Department of Radiation Oncology, H. Lee Moffitt Cancer Center & Research Institute, Tampa, FL, USA

## Abstract

The use of gene expression-based classifiers has resulted in a number of promising potential signatures of patient diagnosis, prognosis, and response to therapy. However, these approaches have also created difficulties in trying to use gene expression alone to predict a complex trait. A practical approach to this problem is to integrate existing biological knowledge with gene expression to build a composite predictor. We studied the problem of predicting radiation sensitivity within human cancer cell lines from gene expression. First, we present evidence for the need to integrate known biological conditions (tissue of origin, RAS, and p53 mutational status) into a gene expression prediction problem involving radiation sensitivity. Next, we demonstrate using linear regression, a technique for incorporating this knowledge. The resulting correlations between gene expression and radiation sensitivity improved through the use of this technique (best-fit adjusted *R*^2^ increased from 0.3 to 0.84). Overfitting of data was examined through the use of simulation. The results reinforce the concept that radiation sensitivity is not driven solely by gene expression, but rather by a combination of distinct parameters. We show that accounting for biological heterogeneity significantly improves the ability of the model to identify genes that are associated with radiosensitivity.

## 1. Introduction

One of the goals of developing biomarkers is for use in patient selection, diagnosis, and management of cancer treatment [1–3]. An important aspect in management of cancer treatment is to understand how a patient will respond to a specific treatment such as radiation therapy. Designing the radiation therapy to maximize cancer cell death is beneficial, and predicting such a response of the cells to radiation therapy is important for effective patient management. Genes such as* RAS* [4, 5] and* p53* [6] have been known to influence the response of tumor cells to radiation treatment. For example,* RAS* has been implicated as a central regulator of radioresistance. Similarly, presence of a mutant* p53* gene is used as an indicator for uncontrolled proliferation of cells, while a wild-type* p53* gene is known to be a tumor suppressor. In addition tissue of origin has been associated with radiosensitivity. For example, the SF2 (survival fraction of cells after 2 Gy of radiation) of melanoma and glioma cell lines has been shown to be higher (radioresistant) than lymphoma and myeloma cell lines [7–9].

The process of developing the systems-based model of radiosensitivity followed a stepwise strategy. The first step was to develop a radiosensitivity classifier to predict cellular radiosensitivity based on gene expression profiles [10]. We developed a multivariable linear regression model that correlated gene expression to radiosensitivity as determined by SF2, in a 35-cell line database. We used a leave-one-out cross-validation approach, where the classifier was developed using 34 of the 35 cell lines as a training set, leaving one cell line as a test set. The basal gene expression profiles and the radiation sensitivity of all 34 cell lines in the training set were used to identify genes that were correlated with radiosensitivity. This was performed using SAM analysis (Significant Analysis of Microarrays) [11] with a false discovery rate of 5%. Genes selected by SAM were then combined as radiosensitivity predictors during the construction of the classifier. A multivariable linear regression model was created using these probesets to predict the SF2 of the test sample and was shown to achieve a statistically significant (*p* = 0.002) predictive accuracy of 62%, within a continuous classification problem. The classifier predicts an actual SF2 value (range: 0.01–1.0) rather than a binary phenotype (radiosensitive versus radioresistant). Importantly, we biologically validated the model by demonstrating that three of the genes selected by the algorithm (*rbap48*,* rgs-19,* and* top-1*) were mechanistically involved in radiation response. Thus, we demonstrated that cellular radiosensitivity is predictable based on gene expression but more importantly we validated this approach as a strategy for the discovery of novel radiosensitivity biomarkers.

Although we had developed a successful mathematical model correlating gene expression and radiosensitivity, we reasoned the model had a number of problems that if overcome would significantly improve its ability to impact the field of radiation biology. First, expansion of the cell line dataset from 35 samples should provide more reliable correlations. Second, there were few genes consistently selected by the classifier. A larger pool of genes would be desirable, as it would allow us to identify the biological networks that regulate cellular radiosensitivity. Third, gene expression was the only variable considered in the model, while there are several biologic factors besides gene expression that are known to influence radiosensitivity. Therefore we focused on strategies aimed at increasing the pool of candidate genes and incorporating biologic variables into the algorithm. One of the advantages of developing the classifier in the NCI-60 is that these cell lines are molecularly well characterized, thus allowing the inclusion of important biological variables into the process. We chose four variables that have been previously correlated to radiation sensitivity: gene expression [10], tissue type [8, 12], RAS mutation status [13–18], and p53 mutation status [19–21]. In addition we expanded the cell line dataset from 35 to 48 cell lines.

## 2. Material and Methods

### 2.1. Microarrays

Gene expression profiles were from Affymetrix HU6800 chips (7,129 genes) from a previously published study [22]. These are publicly available as supplemental data to the published study. The gene expression data had been previously preprocessed using the Affymetrix MAS 5.0 algorithm in average difference units. Negative expression values were set to zero and the chips were normalized to the same mean intensity. Specific cell lines used are listed in Supplemental Table 1 in Supplementary Material available online at https://doi.org/10.1155/2017/6576840.

### 2.2. Radiation Survival Assays (SF2)

The SF2 of cell lines used in model development were previously reported [10, 23]. SF2 values are included in Supplemental Table 1.

### 2.3. Permutation Analysis

Predictions were randomly permutated among cell lines 10,000 times and accuracies greater than or equal to the threshold were counted to calculate a *p* value for significance relative to chance.

### 2.4. Gene Expression Model

Gene expression and radiation sensitivity were described through a linear relationship as described in (1). In this equation, SF2_n_ represents the radiation sensitivity (as measured by SF2) for cell line *n* in the dataset. *k*_*i*_ represents a model coefficient, computed during the training process, and *y*_*ni*_ represents the gene expression value for the *i*th probeset for cell line *n*. The least-squares fit of the individual linear models was compared when selecting probesets of interest for modeling radiosensitivity.

*Gene Expression-Only Model*

(1) $$\begin{array}{c} SF2_{n}=k_{0}+k_{1}\left(\;y_{ni}\;\right). \end{array}$$

### 2.5. Inclusion of Biological Covariates in Model Development

We hypothesized that incorporating biological covariates into the gene selection process would improve the ability of the algorithm to identify radiosensitivity biomarkers. To integrate biological covariates into model development we constructed individual gene-based models using two different equations to relate gene expression and the biological parameters to radiosensitivity (SF2). Specific biological parameters are tissue of origin (TO), RAS mutation status (RAS), and p53 mutation status (p53). 

*Additive Model*

(2) SF2n=k0+k1yni+k2TOn+k3RASn+k4p53n.

*Interactive Model*

(3) SF2n=k0+k1yni+k2TOn+k3RASn+k4p53n+k5yniTOn+k6yniRASn+k7TOnRASn+k8ynip53n+k9TOnp53n+k10RASnp53n+k11yniTOnRASn+k12yniRASnp53n+k13TOnRASnp53n+k14yniTOnRASnp53n⋯.

In (2) and (3), the cell line radiosensitivity (SF2_n_) was modeled as a function of gene expression (*y*) and biological variables (TO, RAS, and p53). Specifically, SF2_n_ represents the radiosensitivity of cell line *n* and *y*_*ni*_ represents the gene expression value of an individual probeset (*i*) for the *n*th cell line in the dataset. A total of 9 different TO values were present in the 48 cell line database. RAS_*n*_ and p53_*n*_ were binary variables (wild-type/mutated) for the *n*th cell line. Thus, the additive model considered a total of 13 terms (an intercept, gene expression, 9 TO, RAS, and p53). The more complex interactive model initially considered all possible terms and 2-, 3-, and 4-way interactions among these terms. Without accounting for linearly dependent terms, there are 180 terms total, far more than the number of observations (48). These include an intercept, 14 terms involving a single variable (gene expression, 9 TO, 2 p53, and 2 RAS), 53 paired terms, 76 triples, and 36 terms with four variables interacting.

While the equations represent models with very large number of variables, the number of nonsingular terms was far less due to the small sample size. Additionally, linearly dependent variables (typically interactions with no examples present) are dropped from the model. Interactions of larger numbers of variables were dropped in favor of fewer in the case of linearly dependent variables. Thus there are only 29 terms in the linear model (an intercept, gene expression, 9 TO, p53, RAS, 15 two-way interactions, and 2 three-way interactions) (Table 1). A gene-based linear model was constructed for each gene (7168 probesets), correlating expression and biological parameters with the measured SF2 using a least-squares fit. We compared the sum squared error of the gene expression-based linear models to the null model, consisting of biological parameters and no expression (SSE = 1.2).

### 2.6. Random Variables

Random variables for exploring the effect of* RAS* and* p53* mutation status were created and uniformly distributed into two states (one each for the mutated and wild-type status). The frequencies of these states were similar to the true distributions in the data. Similarly, a random variable was defined for TO, with each sample being assigned a tissue type at random. This new dataset with randomly assigned biological parameters was used to test whether the improvement in linear fit achieved by both the additive and interactive model was due to the integration of biological variables or due to chance.

## 3. Results

### 3.1. Expansion of Cell Line Dataset Lowers Classification Accuracy

**Figure 1:**
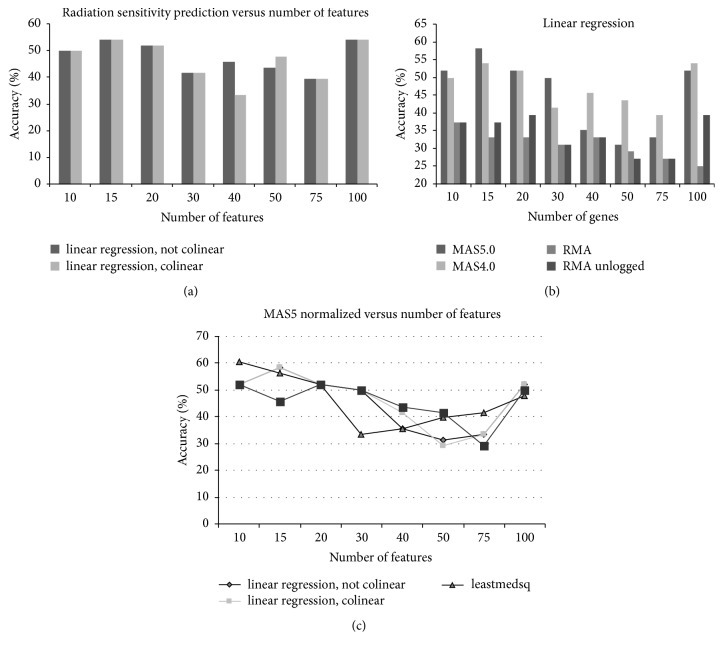
Investigation of building predictors for radiation sensitivity in 48 cell lines. (a) Classification accuracy of radiation sensitivity predictor built from 48 cell lines, using different numbers of features in the regression model. (b) Classification accuracy of radiation sensitivity predictor built from 48 cell lines, using different types of normalization. MAS5.0 and MAS4.0 algorithms generated the most accurate predictors. (c) Classification accuracy of radiation sensitivity predictor built from 48 cell lines, using different types of classification algorithms, including linear regression, least median, and SMO.

As described above we previously developed a gene expression radiosensitivity classifier [10] as a continuous prediction rather than a binary classification problem (i.e., radiosensitive versus radioresistant). During development of the model we had observed that increasing the number of samples increased the classifier accuracy (data not shown). Thus we hypothesized that increasing the cell line dataset to 48 cell lines would result in a more accurate model. Surprisingly, the classifier technique was not as accurate when the cell line population was increased to 48 (compared to 35) cell lines. The best linear regression-based classifier using the 48 cell lines correctly classified 26/48 samples (54%) (Figure 1(a)) compared to 25/35 (71%) for the best classifier in the 35-cell line dataset. We explored the use of alternate normalization (Figure 1(b)); however the maximum accuracy was 28/48 or 58%. Additionally, we looked at alternate predictors (Figure 1(c)) but the decreased accuracy in the 48-cell line dataset was consistent. Although the results were still statistically significant in that the classifier in the 48 cell line dataset performed better than chance (*p* = 0.0094), we were interested in understanding the reason for the decreased accuracy.

### 3.2. Understanding the Influence of Confounding Factors

**Figure 2:**
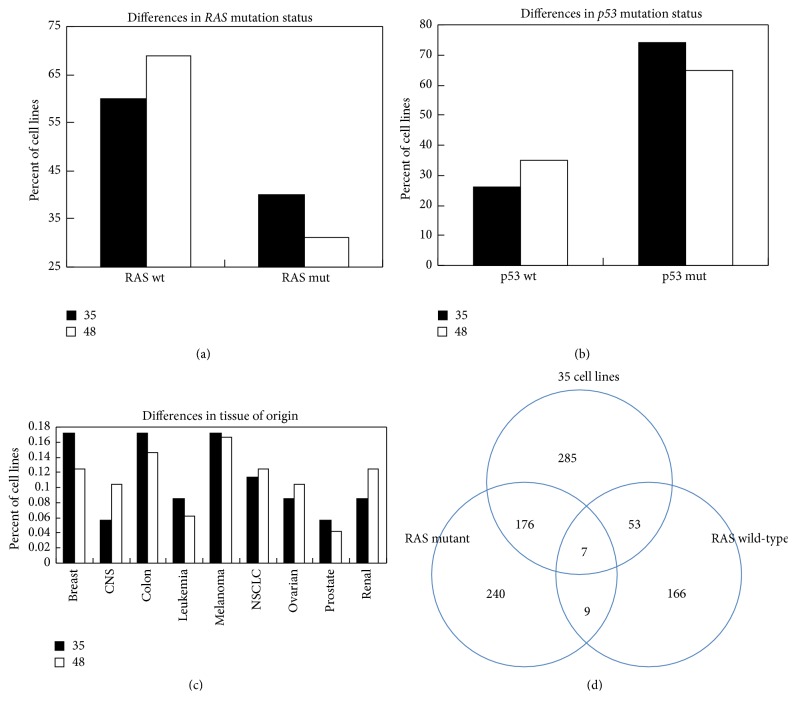
Biological characteristics differ when considering 35 cell lines versus an expanded set of 48 cell lines. (a) The proportion of RAS wild-type cell lines increased (60% to 69%). (b) The proportion of p53 wild-type cell lines increased (26% to 35%). (c) Tissue of origin of cell lines did not change significantly. (d) Venn diagram showing the lack of concordance in correlation when using a test for correlation (*p* < 0.05) using only RAS mutant or RAS wt cell lines in the 35-cell line set. Only 16 probesets were found correlated in both sets.

The decrease in classification accuracy suggested that the linear regression model based only on gene expression data did not fully represent the classification problem. We hypothesized that accounting for the biological diversity of cell lines in the database would be of importance. Several biological variables available for the NCI-60 cell lines include tissue of origin (TO),* RAS* mutational status (wt/mut) (RAS), and* p53* mutational status (p53). These variables have been implicated in the biological regulation of radiation sensitivity [13, 24]. Among the 48 cell lines, the* RAS*-mutated cell lines represent only 31% (15/48) of cell lines whereas they represented 40% (14/35) in the 35-cell line database (Figure 2(a)). The* p53* mutation status was also different between the two groups; 26 cell lines were* p53* mutants in the 35 cell lines; however only 5 additional mutants were added, changing the proportions from 74% down to 65% of the cell line population (Figure 2(b)). Tissue of origin was similar in proportions in the two groups (Figure 2(c)). Since only one additional* RAS*-mutated cell line was added when increasing the dataset to 48 we first focused on determining if* RAS* mutation status impacted the gene selection process.

The oncogenic protein RAS has been proposed to mediate a central mechanism in radiation resistance [16]. We tested whether the presence of a RAS mutation, which usually affords a chronically active RAS protein, was an important source of variability within the dataset. This was done by determining whether the genes selected by the 35 cell line classifier were dependent or independent of RAS status. We stratified the original 35 cell lines by RAS status and performed the gene selection step (correlation of gene expression and SF2) in each group of cell lines. The three genes (rbap48, rgs-19, and r5pia) selected by the original classifier (without RAS stratification) were previously shown to be highly useful in predicting radiosensitivity. These genes were highly ranked among the RAS-mutated cell lines but not in the wild-type lines, suggesting that the RAS-mutated cell lines were driving the classification process. RbAp48, rgs-19, and r5pia were ranked 19th, 46th, and 262nd out of 7,129 probesets by *R*2 values from the RAS-mutated cell lines. In wild-type cell lines, these same genes are ranked 743rd, 758th, and 397th, respectively. Interestingly, these three genes ranked in the top 10 genes when all cell lines were considered together (5th, 1st, and 9th) (Table 2). These results suggest that the biological diversity of cell lines studies (e.g., RAS-mutated and RAS wt) can significantly impact the evaluation of genes with respect to outcomes. In particular, two diverse biological types mixed in different proportions can lead to highly variable ranking as demonstrated by our 35-cell line experiment.

### 3.3. Integrating Biological Covariates

As a result of the analysis of confounding factors, three variables (TO, RAS, and p53) were integrated in the gene expression analysis using two approaches: an additive model and an interaction-based linear model. The gene selection process was repeated using these approaches on the 48 cell lines. RAS and p53 status indicators were binary variables that indicate wild-type (wt) or mutational (mut) status of the gene for a cell line. The indicator for tissue of origin (TO) has 9 levels, one for each type of tissue from which the tumor cell line originated [22]. The analysis was performed for each probeset and the model fit parameter adjusted-*R*^2^ (Adj-*R*^2^) was used to determine if the model improved by inclusion of the covariates. The adjusted-*R*^2^ was used instead of *R*^2^ in these experiments to adjust for addition of regressors in the equations.

**Figure 3:**
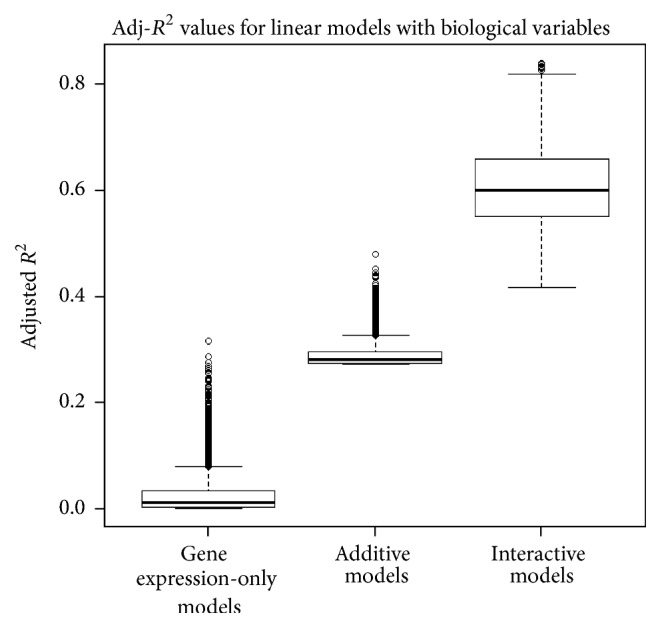
Adj-*R*^2^ values for linear equations fitting SF2 on 48 cell lines. Adj-*R*^2^ values increase systematically as more covariates are included in the linear model.

Figure 3 shows a box plot summarizing the Adj-*R*^2^ values from all probeset models individually when correlated with radiation response (SF2) in the 48-cell line database. In the gene expression-only model, fewer probesets had a model fit better than 0.2 (<30 of the 7129 probesets), with the best fit being just above 0.3. The average fit for the additive model was 0.28 with a maximum fit of 0.48. With the interactive model, the average fit improved to 0.6 with a maximum value of 0.84. Thus the integration of biological variables, including all interactions, improved the modeling fit considerably.

### 3.4. Verification of Model Fit

The improvement in Adj-*R*^2^ of a linear model could be attributed simply to the addition of more variables in the model (e.g., overfitting) [25]. We compared the fit of the expanded linear models to the fit obtained using variables with randomly generated values. Random variables that do not have any meaningful information and are uncorrelated to the outcome are expected to produce models with lower Adj-*R*^2^ values.

Table 3 shows the change in the model fit (Δ*R*^2^) when terms are added to a linear model. Both the change in fit from biological indicators and randomly generated variables are recorded. For each biological covariate (RAS, p53, and TO), inclusion of the variable in an additive model does not improve the model fit more than including randomly generated variables. The inclusion of TO in the additive model provides no more information than would be expected by chance (average change in *R*^2^: TO 0.254, random 0.256). Even with the addition of multiple terms, the additive model improves no better than by chance. When gene expression, TO, and RAS are combined in the additive model, the correlation of the model improves by 0.256. However, the same improvement is observed when the random variable is added (Δ*R*^2^ = 0.257).

The difference between including biological variables and random variables in the interaction-based models is more significant. For example, the change in *R*^2^ for the additive model using RAS, TO, and gene expression was similar to that of random variables; however in the interaction model, the correlation improves by 0.272 whereas the interaction of random variables (for TO and RAS) drops by 0.213 (Δ*R*^2^ = −0.213). When including all three terms in the interaction models, the Adj-*R*^2^ improves by 0.317 but the random variables cause a drop in correlation (Δ*R*^2^ = −0.103).

**Figure 4:**
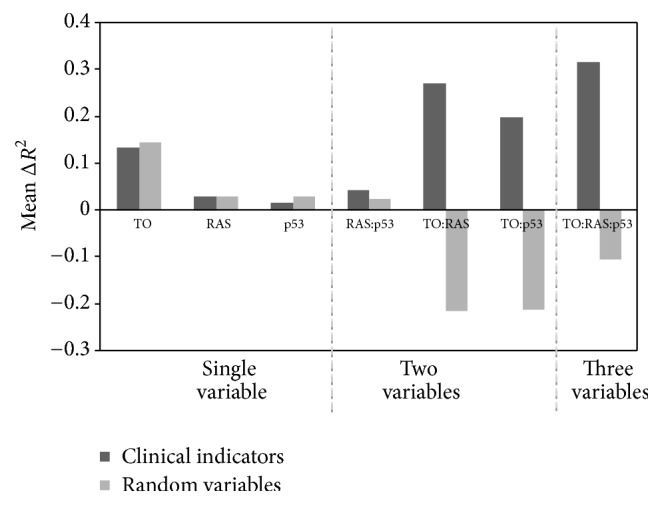
Change in Adj-*R*^2^ values obtained by incorporating interaction terms in linear model for either biological indicators or random variables. Linear models were created for each gene, incorporating the three biological indicators. Differences in Adj-*R*^2^ values were computed for each experiment between using an additive model and an interaction-based model. The mean difference in *R*^2^ was recorded when adding interactions terms to the individual linear models.

Figure 4 summarizes the trend that when two or more biological variables are considered, this results in better linear models than expected from randomly generated variables. The interaction of random variables with gene expression data alone provides a marginal improvement in the fit; however, when two or more random variables interact, the lack of information in each variable translates into poorer fit of the linear model to the radiation sensitivity outcome. In contrast, the interaction of the biological variables adds more information to the linear model, as shown by the improvement in Adj-*R*^2^ values in Table 3 and Figure 4.

## 4. Discussion

The central aim of our research efforts is the development of a systems biology-based understanding of the biological networks that regulate radiosensitivity. In systems biology, central biological processes are proposed to be organized as complex and redundant networks with complex interactions within and across different biological scales (molecular, network/pathway, cellular, tissue, and organism) [26, 27]. A central requirement in systems biology modeling is the development of mathematical approaches to relate biological scales. In a previous study we established a linear regression algorithm as a valid biological approach to relate gene expression and radiosensitivity within a 35-cell line dataset [10]. However, a problem with this initial modeling approach was that gene expression by itself resulted in the identification of very few genes out of the 7,168 probesets evaluated. Therefore a better approach to gene selection was required in order to model the networks that regulate radiosensitivity.

In this study we show that accounting for biological confounders within a linear regression model of radiosensitivity significantly improves the ability of the algorithm to fit gene expression and radiosensitivity, which resulted in a better ability to identify significant genes. We showed that simply adding the biological variables did not improve the fit more than what was expected from chance but when a more complex interaction-based model was utilized, its performance was superior to chance.

Although unexpected, the previously developed predictor of radiation sensitivity performed much worse when the cell line set was expanded to 48 cell lines. The underlying cause of this difference was determined to be from shifting proportions of cell lines with key biological characteristics that have been previously implicated in modulating radiation response. Specifically, we show that* RAS*-mutated cell lines had a large impact in gene selection in the 35-cell line dataset. Three of the top genes selected by the 35-cell line classifier were highly ranked by* RAS*-mutated cell lines but not by* RAS* wt cell lines. However when the model was expanded to 48 cell lines, the impact of these cell lines was diluted with the addition of predominantly* RAS* wt cell lines. Once these factors were accounted for within the modeling process, genes were identified as related to radiation sensitivity most significantly through interactions with these biological characteristics. This work led to the development of a systems-based predictive model of tumor intrinsic radiosensitivity that was validated in three independent clinical cohorts of patients treated with chemoradiotherapy [28]. However, a key insight into this process was the identification and incorporation of confounders.

The strategy presented here may have applications in the development of clinical predictive/prognostic models. For example, we have already shown that this process led to development of a predictive model of intrinsic radiosensitivity that has been clinically validated [23, 28–35]. However, clinical cohorts very often present similar diversity as that represented in the cell line database utilized and identifying key biological covariates and a mathematical approach to account for them might significantly enhance our ability to develop predictive models with clinical utility.

The inclusion of biological variables significantly improved the ability of most genes to describe the relationship between gene expression and radiation response in a linear regression model. However, the inclusion of additional parameters and their interactions within the same equation almost certainly leads at least in some instances to overfitting. It is important to note that the selection process of genes for further validation (e.g., by choosing the best-fit genes) does not require overfitting to be completely removed. Rather, it is expected that overfitting will uniformly increase the fit of genes with radiation sensitivity. In addition, the behavior of the random variables in the interaction models clearly indicates that the biological variables do provide meaningful information, and rather than causing overfitting of the model to the data, the biological variables can be used to create a better model for gene selection.

Finally, an improvement in linear fit should be similarly obtained when adding a randomly generated variable into the model instead of a variable that carries biological significance. However, it is intriguing that not all variables considered had similar impact in improving the model, as might be expected due to chance. For example* RAS* was significantly more important than* p53* in improving the model. This observation suggests that at least part of the improvement obtained by the expanded linear models is due to a better representation of biology.

## 5. Conclusion

In conclusion, we demonstrate that incorporating biological covariates into a gene expression model of tumor intrinsic radiosensitivity can improve the modeling process. Accounting for biological heterogeneity can identify genes that are associated with radiosensitivity, which in turn led to the development of a successful model of clinical response to radiotherapy [23, 28–35].

## Supplementary Material

Supplemental Table 1: Cell Lines used in the experiments.

## Figures and Tables

**Table 1:** Terms used in linear modeling. The term (*y*) represents gene expression. The operator × represents an interaction term between two or more variables.

Terms	Terms
Intercept	*y* × tissueTypeBREAST
*y* (gene expression)	*y* × tissueTypeCNS
tissueTypeBREAST	*y* × tissueTypeCOLON
tissueTypeCNS	*y* × tissueTypeLEUK
tissueTypeCOLON	*y* × tissueTypeMELAN
tissueTypeLEUK	*y* × tissueTypeNSCLC
tissueTypeMELAN	*y* × tissueTypeOVAR
tissueTypeNSCLC	*y* × tissueTypePROSTATE
tissueTypeOVAR	*y* × RASmut
tissueTypePROSTATE	*y* × p53mut
RASmut	tissueTypeBREAST × RASmut
p53mut	tissueTypeCOLON × RASmut
	tissueTypeMELAN × RASmut
	tissueTypeNSCLC × RASmut
	tissueTypeOVAR × RASmut
	*y* × tissueTypeBREAST × RASmut
	*y* × tissueTypeCOLON × RASmut

**Table 2:** Ranking of previously validated radiosensitivity genes when considering all cell lines (*n* = 35), RAS-mutated cell lines only (*n* = 14), and RAS wt cell lines only (*n* = 21). Significant differences in ranking occur when considering the biological variable of RAS mutation status.

Gene	Overall ranking	RAS-mutated cell line	RAS wt cell lines
rbap48	5	19	743
rgs-19	1	46	758
r5pia	9	262	397

**Table 3:** Change in Adj-*R*^2^ value obtained by adding terms and complexity to the linear model. Results obtained with clinical indicators TO, RAS, and p53 are compared to Adj-*R*^2^ values obtained using random variable for each indicator.

Model terms	Model comparison	Mean Δ*R*^2^ value	
		Clinical indicators	Random variables
GeneEx : TO	GenEx only versus additive	0.254	0.256
	Additive versus interaction	0.134	0.146

GeneEx : RAS	GenEx only versus additive	0.060	0.004
	Additive versus interaction	0.030	0.031

GeneEx : p53	GenEx only versus additive	0.026	0.0007
	Additive versus interaction	0.016	0.031

GeneEx : TO : RAS	Basic versus additive	0.256	0.257
	Additive versus interaction	0.272	*−0.213*

GeneEx : TO : p53	Basic versus additive	0.262	0.257
	Additive versus interaction	0.198	*−0.211*

GeneEx : RAS : p53	Basic versus additive	0.062	0.022
	Additive versus interaction	0.042	0.024

GeneEx : TO : RAS : p53	Basic versus additive	0.265	0.258
	Additive versus interaction	0.317	*−0.103*
