# Supplementary material for: Integrating Biological Covariates into Gene Expression-Based Predictors of Radiation Sensitivity
Source: Int J Genomics. 2017 Feb 8;2017:6576840. doi: 10.1155/2017/6576840 (PMC5320380; doi:10.1155/2017/6576840)
Supplement: Supplementary file 1 — Supplemental Table 1: Cell Lines used in the experiments. [file 6576840.f1.pdf]

Supplemental Table 1: Cell Lines used in the experiments.

Cell Line	Recorded SF2	Original 35 cell line set	Expanded 48 cell line set
BREAST_BT549	0.632	yes	yes
BREAST_HS578T	0.79	yes	yes
BREAST_MDAMB231	0.82	yes	yes
BREAST_T47D	0.52	yes	yes
CNS_U251	0.57	yes	yes
COLON_COLO205	0.69	yes	yes
COLON_HCT116	0.38	yes	yes
COLON_HCT15	0.4	yes	yes
COLON_HT29	0.79	yes	yes
MELAN_M14	0.42	yes	yes
MELAN_MALME3M	0.8	yes	yes
MELAN_SKMEL2	0.66	yes	yes
MELAN_SKMEL28	0.74	yes	yes
NSCLC_A549ATCC	0.61	yes	yes
NSCLC_H460	0.84	yes	yes
OVAR_OVCAR3	0.55	yes	yes
OVAR_OVCAR5	0.408	yes	yes
PROSTATE_DU145	0.52	yes	yes
PROSTATE_PC3	0.484	yes	yes
RENAL_A498	0.61	yes	yes
RENAL_ACHN	0.72	yes	yes
RENAL_CAKI1	0.37	yes	yes
BREAST_MCF7	0.576	yes	yes
BREAST_MDAMB435	0.1795	yes	yes
CNS_SF539	0.82	yes	yes
COLON_KM12	0.42	yes	yes
COLON_SW620	0.62	yes	yes
LEUK_CCRFCEM	0.185	yes	yes
LEUK_HL60	0.315	yes	yes
LEUK_MOLT4	0.05	yes	yes
MELAN_LOXIMVI	0.68	yes	yes
MELAN_SKMEL5	0.72	yes	yes
NSCLC_HOP62	0.164	yes	yes
NSCLC_NCIH23	0.086	yes	yes
OVAR_SKOV3	0.9	yes	yes
RENAL_SN12C	0.62		yes
CNS_SF268	0.45		yes
CNS_SNB19	0.43		yes
CNS_SNB75	0.55		yes
COLON_HCC-2998	0.44		yes
MELAN_UACC257	0.48		yes
MELAN_UACC62	0.52		yes
NSCLC_EKVX	0.7		yes
NSCLC_HOP92	0.43		yes
OVAR_OVCAR4	0.29		yes
OVAR_OVCAR8	0.6		yes
RENAL_7860	0.66		yes
RENAL_UO31	0.62		yes
